# Algorithm of face anti-spoofing based on pseudo-negative features generation

**DOI:** 10.3389/fnins.2024.1362286

**Published:** 2024-04-12

**Authors:** Yukun Ma, Chengzhen Lyu, Liangliang Li, Yajun Wei, Yaowen Xu

**Affiliations:** ^1^School of Software, Henan Institute of Science and Technology, Xinxiang, China; ^2^School of Information Engineering, Henan Institute of Science and Technology, Xinxiang, China; ^3^School of Information and Electronics, Beijing Institute of Technology, Beijing, China; ^4^Data and AI Technology Company, China Telecom Corporation Ltd., Beijing, China

**Keywords:** face anti-spoofing, pseudo-negative feature, features generation, feature analysis, cross-domain

## Abstract

**Introduction:**

Despite advancements in face anti-spoofing technology, attackers continue to pose challenges with their evolving deceptive methods. This is primarily due to the increased complexity of their attacks, coupled with a diversity in presentation modes, acquisition devices, and prosthetic materials. Furthermore, the scarcity of negative sample data exacerbates the situation by causing domain shift issues and impeding robust generalization. Hence, there is a pressing need for more effective cross-domain approaches to bolster the model’s capability to generalize across different scenarios.

**Methods:**

This method improves the effectiveness of face anti-spoofing systems by analyzing pseudo-negative sample features, expanding the training dataset, and boosting cross-domain generalization. By generating pseudo-negative features with a new algorithm and aligning these features with the use of KL divergence loss, we enrich the negative sample dataset, aiding the training of a more robust feature classifier and broadening the range of attacks that the system can defend against.

**Results:**

Through experiments on four public datasets (MSU-MFSD, OULU-NPU, Replay-Attack, and CASIA-FASD), we assess the model’s performance within and across datasets by controlling variables. Our method delivers positive results in multiple experiments, including those conducted on smaller datasets.

**Discussion:**

Through controlled experiments, we demonstrate the effectiveness of our method. Furthermore, our approach consistently yields favorable results in both intra-dataset and cross-dataset evaluations, thereby highlighting its excellent generalization capabilities. The superior performance on small datasets further underscores our method’s remarkable ability to handle unseen data beyond the training set.

## 1 Introduction

With the continuous development of computer technology, identity authentication based on face information has been widely used. However, most existing face recognition methods are very vulnerable to face prosthesis attacks. Face spoofing attack refers to illegal users attempting to cheat the face authentication system and the face detection system through some prosthesis methods, such as print attacks, replay attacks, and mask attacks. Face anti-spoofing is developed to detect illegal facial spoofing attacks, thereby improving the security of face authentication systems (Yu et al., 2022).

Though facial recognition technology has been widely used in biometric authentication, it is susceptible to presentation attacks (commonly referred to as “spoofing attacks”), which have attracted much attention in secure scenarios. These attack forms include using synthesized or fake facial images or information to mimic the facial features of legitimate users, thereby bypassing facial recognition systems. Examples of such attacks include printed photos, facial digital images on electronic screens, 3D masks, and other innovative methods. There are special material attacks, where facial models made from special materials attempt to evade traditional facial recognition systems; meanwhile, virtual generation attacks utilize computer graphics and generative adversarial networks (GANs) to produce realistic synthetic faces and bypass facial recognition systems; additionally, lighting manipulation attacks use lighting effects, special lights, or reflective materials to change facial appearance, making it challenging for systems to accurately identify faces. Though various methods have been proposed to defend against these attacks, existing defense methods often lack sufficient generalization ability when confronted with unknown attacks types (de Freitas Pereira et al., 2013). In practical scenarios, training facial anti-spoofing models to predict all types of attacks is a challenging task.

Face anti-spoofing technology, designed to detect and prevent fraud in facial recognition, has significantly advanced in recent years, yielding promising results. However, a major challenge for current methods is their limited ability to generalize to previously unseen or novel attack types. In the real world, it’s nearly impossible to anticipate and incorporate all potential attack scenarios into the training phase, which makes maintaining effectiveness difficult.

As technology evolves and face anti-spoofing techniques become more sophisticated, attackers are also adapting their deceptive methods, leading to new and more complex attack forms. The vast and diverse data space associated with prosthetic attacks, involving high-quality masks or other facial replicas, poses a significant challenge for cross-domain face anti-spoofing. This diversity in attack methods, coupled with variations in presentation, acquisition devices, and prosthetic materials, complicates the task of developing robust and generalizable solutions.

In cross-domain scenarios, where data from multiple sources or domains are involved, existing methods often face significant challenges in training and testing across various devices and materials. These introduce distinct characteristics and variations that can greatly impact model performance and reliability. The fundamental issue is the inadequacy of negative sample data when faced with diverse attacks or perturbations. This scarcity prevents models from adequately learning and generalizing to new, unseen domains, leading to domain shift issues during learning. There’s an urgent need for more robust and effective approaches to address these issues and enhance cross-domain performance.

The contributions of this paper are numerous and significant. Firstly, we introduce an innovative algorithm capable of generating pseudo-negative features by collecting and analyzing features from existing datasets. Secondly, we employ the Kullback–Leibler (KL) divergence loss function to effectively guide the distribution of the generated virtual features, ensuring their alignment with the desired characteristics and further optimizing the system’s accuracy. Finally, our approach has achieved promising results across multiple cross-domain tests, demonstrating robust performance. Overall, our contributions advance the state-of-the-art in face anti-spoofing technology.

## 2 Related work

At the initial stage, manually annotated features were used to construct face anti-spoofing. Määttä et al. (2011) developed a method based on the analysis of facial textures to determine whether there is a living person or facial imprint in front of the camera. de Freitas Pereira et al. (2014) extracted local binary patterns (LBP) features in three orthogonal planes of spatiotemporal space for face fraud detection. Similarly, most of the histogram-based 2D features can be generalized to their corresponding 3D forms. In recent years, face anti-spoofing based on deep learning has attracted much attention. Compared with traditional hand-crafted features, deep features learned by the neural network have a more robust representation ability, and the accuracy of the trained model is also greatly enhanced. Yang et al. (2014) first applied the Convolutional Neural Network (CNN) to face anti-spoofing by using the AlexNet network model as a feature extractor to extract the features of the original image and using the Support Vector Machine (SVM) for classification. Menotti et al. (2015) employed the hyperparameter search method to find a suitable CNN network structure for face fraud detection. To narrow the search range of hyperparameters, the searched CNN contained at most three convolutional layers. Rehman et al. (2017) trained an 11-layer VGG network and two variant networks in an end-to-end manner for face fraud detection. Nagpal and Dubey (2019) investigated deeper face fraud detection based on ResNet and GoogLeNet. Li et al. (2016) used transfer learning to extract features after fine-tuning the pre-trained VGG face model, which mitigated overfitting in the model. Some researchers replaced the original hand-crafted features with features learned by the network (Cai et al., 2022). Additionally, the optical flow feature provides an effective method for extracting motion information from videos (Simonyan and Zisserman, 2014; Sun et al., 2016, 2019). Yin et al. (2016) found motion cues of face fraud based on optical flow features. Pinto et al. (2015) proposed a feature based on low-level motion features and mid-level visual encoding for face fraud detection. De Marsico et al. (2012) extracted geometrically invariant features around facial feature points to detect cues in video replay. Moreover, some studies used temporal features between consecutive frames for face anti-spoofing (Wang et al., 2022a).

In the early stage, the deep learning-based detection algorithm employed the softmax loss function for face authenticity classifications. Although these methods improved the detection performance on a single database, their generalization ability remained challenging when tested across data sets. Different from the previous binary classification approach, Liu et al. (2018) proposed training networks using auxiliary information. This method combined face depth information and rPPG (remote photoplethysmography) as an auxiliary supervised guidance model to learn essential features, and it achieved a good detection effect. Kim et al. (2019) introduced reflection-based supervision based on depth graph supervision, which further improved the network’s detection performance. Moreover, Li et al. (2020) and Yu et al. (2020) proposed new convolution operators and loss functions for live face detection, respectively. To better resist various unknown attacks and improve the generalization ability of deep models across data sets, researchers also used zero-shot learning (Liu et al., 2019), domain adaptation, and domain generalization to enhance the model’s generalization ability (Saha et al., 2020; Wang et al., 2021). To obtain better domain generalization approaches, Jia et al. (2020) proposed an end-to-end single-side domain generalization framework (SSDG) to improve the generalization ability of face anti-spoofing. Furthermore, Dong et al. (2021) proposed an end-to-end open-set face anti-spoofing (OSFA) approach for recognizing unseen attacks. However, the accuracy and generalization ability of classification models are still areas of active research.

In recent years, the application of transformers in the visual domain has led to numerous advancements in addressing domain generalization issues. Specifically, approaches like the Domain-invariant Vision Transformer (DiVT) have effectively leveraged transformers to enhance the generalization capabilities of face anti-spoofing tasks (Liao et al., 2023). Additionally, initializing Vision Transformers (ViT) with pre-trained weights from multimodal models such as CLIP has been shown to improve the generalization of FAS tasks (Srivatsan et al., 2023). Furthermore, adaptive ViT models have been introduced for robust cross-domain face anti-spoofing (Huang et al., 2022). By employing overlapping patches and parameter sharing within the ViT network, these approaches efficiently utilize multiple modalities, resulting in computationally efficient face anti-spoofing solutions (Antil and Dhiman, 2024).

To further enhance domain generalization, unsupervised or self-supervised methods have been employed during model construction and training. One such approach involves stylizing target data to match the source domain style using image translation techniques and then classifying the stylized data using a well-trained source model (Zhou et al., 2022a). Additionally, novel frameworks such as Source-free Domain Adaptation for Face Anti-Spoofing (SDAFAS; Liu et al., 2022a) and a source data-free domain adaptive face anti-spoofing framework (Lv et al., 2021) have been proposed to tackle issues related to source knowledge adaptation and target data exploration in a source-free setting. These frameworks aim to optimize the network in the target domain without relying on labeled source data by treating it as a problem of learning with noisy labels.

Moreover, a new perspective for domain generalization in face anti-spoofing has been introduced that focuses on aligning features at the instance level without requiring domain labels (Zhou et al., 2023). Frameworks like the Unsupervised Domain Generalization for Face Anti-Spoofing (UDGFAS) exploit large amounts of easily accessible unlabeled data to learn generalizable features (Liu et al., 2023), thereby enhancing the performance of FAS in low-data regimes. These approaches explore the relationship between source domains and unseen domains to achieve effective domain generalization.

Additionally, a self-domain adaptation framework has been proposed that leverages unlabeled test domain data during inference time (Wang et al., 2021). Another approach involves encouraging domain separability while aligning the live-to-spoof transition (i.e., the trajectory from live to spoof) to be consistent across all domains (Sun et al., 2023). The Adaptive Mixture of Experts Learning (AMEL) framework (Zhou et al., 2022b) exploits domain-specific information to adaptively establish links among seen source domains and unseen target domains, further improving generalization. A generalizable Face Anti-Spoofing approach based on causal intervention is proposed, aiming to enhance the model’s generalization ability in unseen scenarios by identifying and adjusting domain-related confounding factors (Liu et al., 2022b).

Studying the local features of images has also proven beneficial for achieving good domain generalization. For instance, PatchNet reformulates face anti-spoofing as a fine-grained patch-type recognition problem, recognizing combinations of capturing devices and presentation materials based on patches cropped from non-distorted face images (Wang C. Y. et al., 2022). Furthermore, a novel Selective Domain-invariant Feature Alignment Network (SDFANet) has been proposed for cross-domain face anti-spoofing. This network aims to seek common feature representations by fully exploring the generalization capabilities of different regions within images (Zhou et al., 2021).

The current limited cross-domain performance of facial liveness detection methods is due to the incomplete nature of negative sample data under diverse attacks. Based on the above research, considering that the existing feature information is not complete while disregarding the relationship between features, this paper proposes a new face anti-spoofing method based on CNN to generate pseudo-negative feature data of the training sample, and then calculate the feature distribution, and control the generation of the virtual feature distribution by using the KL divergence loss function. Additionally, based on the generated new pseudo data, the proposed method employs a collaborative training algorithm with the original features to improve the generalization performance of face anti-spoofing systems.

## 3 Proposed method

**Figure 1:**
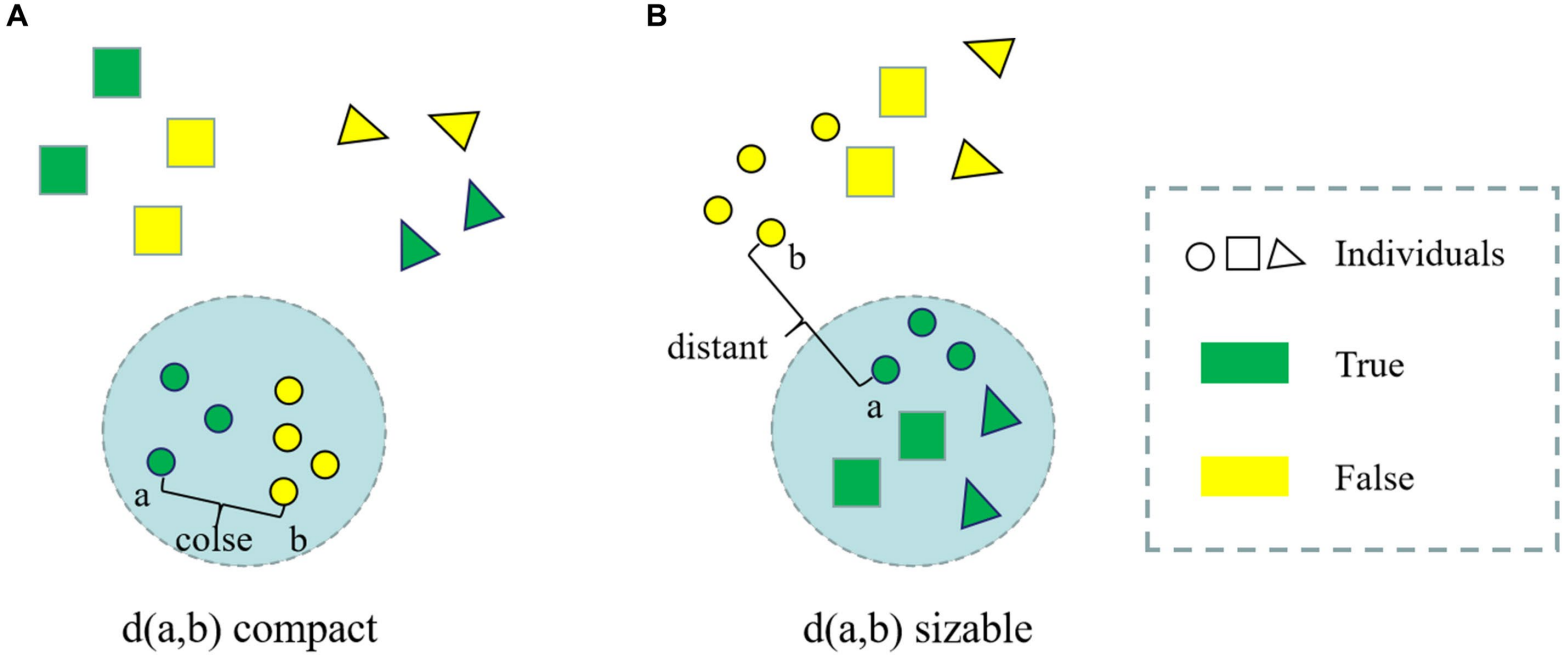
**(A)** True and false samples of different people in human vision; **(B)** True and false samples of different people in the living body detection classifier.

Face anti-spoofing is a binary classification task (real/fake). Unlike typical coarse-grained binary classification tasks, the liveness detection task exhibits a property that is inconsistent with human visual distance, as illustrated in Figure 1.

Currently, most of the studies on face anti-spoofing systems focus on increasing the type and number of attack samples to enhance the stability and generalization of face anti-spoofing systems. However, due to the unseen data in the training stage, the original method has some limitations in dealing with unknown attack methods.

By analyzing existing face anti-spoofing methods, it is observed that the incompleteness of negative samples is the primary factor limiting the algorithm’s cross-domain performance. Therefore, this method aims to research pseudo-negative sample features, expand the training dataset, and improve the cross-domain generalization of face anti-spoofing methods. First, to address the issue of incomplete negative samples, this study generates pseudo-negative features based on the distribution of *bona fide* and attack features. These features complement existing negative class data, enhancing the diversity and completeness of the negative sample dataset. Then, this study uses pseudo-negative features together with existing negative class data to assist in training a feature classifier for real faces, further adjusting the parameters of the feature extractor. The generation of pseudo-negative features leads to more comprehensive negative sample features during training, making the system cover attack data in a broader range of scenarios and thus improving the generalization of the detection method.

In the context of prosthetic attacks, there exists a certain level of feature dispersion across various attack scenarios, suggesting a wider intra-class variation. Due to this, cross-scenario liveness detection poses a certain challenge, and collecting all types of attack data during the training process can be challenging. The differences in intra-class distribution between seen and unseen attack types often lead to domain shift issues. To tackle these challenges, this study employs a technique for generating pseudo-negative class features, aiming to directly learn the mapping between the visual space of images and the semantic space of features. This method can avoid information loss. Finally, this study develops an end-to-end training model applicable to cross-domain face liveness detection.

**Figure 2:**
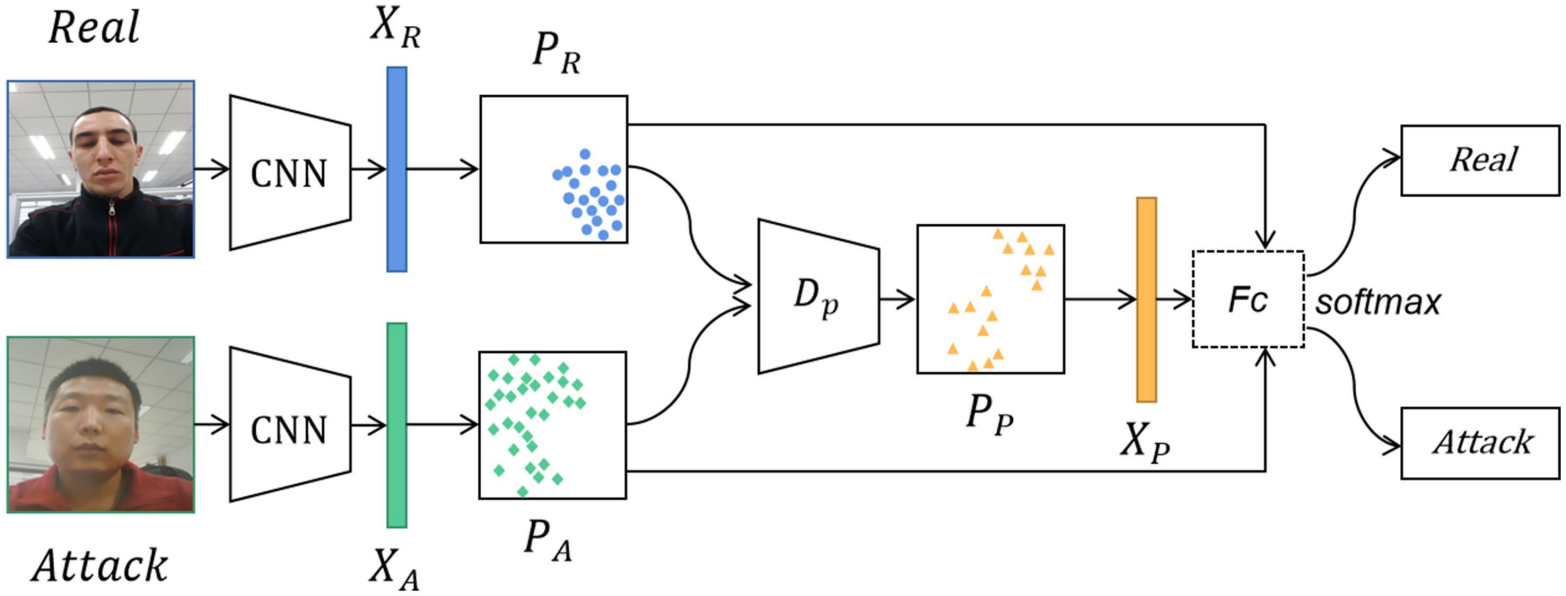
The structure diagram of generating pseudo-negative features for face anti-spoofing. The real and attack images are input into the CNN to extract the *bona fide* features and attack features. Then, the distribution of the attack features and the distribution of the *bona fide* features are obtained. These two feature distribution data are fed into the pseudo-negative feature generator to generate the distribution of pseudo-negative features. Finally, the classification task is completed by going through the Fc and the softmax layers. Facial images reproduced with permission from OULU-NPU dataset (Boulkenafet et al., 2017a).

The method proposed in this paper comprises of feature analysis, feature generation, and collaborative training. As illustrated in Figure 2, the general workflow of the method is as follows: First, after images are inputted, the CNN generates multi-dimensional feature tensor data from the training samples. Then, the tensor data is analyzed to generate new feature data based on their feature distribution and KL divergence value. Meanwhile, attack types and unseen data from the training stage are incorporated to augment the original set of negative features. Finally, the model is trained using both virtual and existing sample features, allowing us to gather the feature distribution of *bona fide* samples and subsequently improve the accuracy and robustness of live face detection.

**Figure 3:**
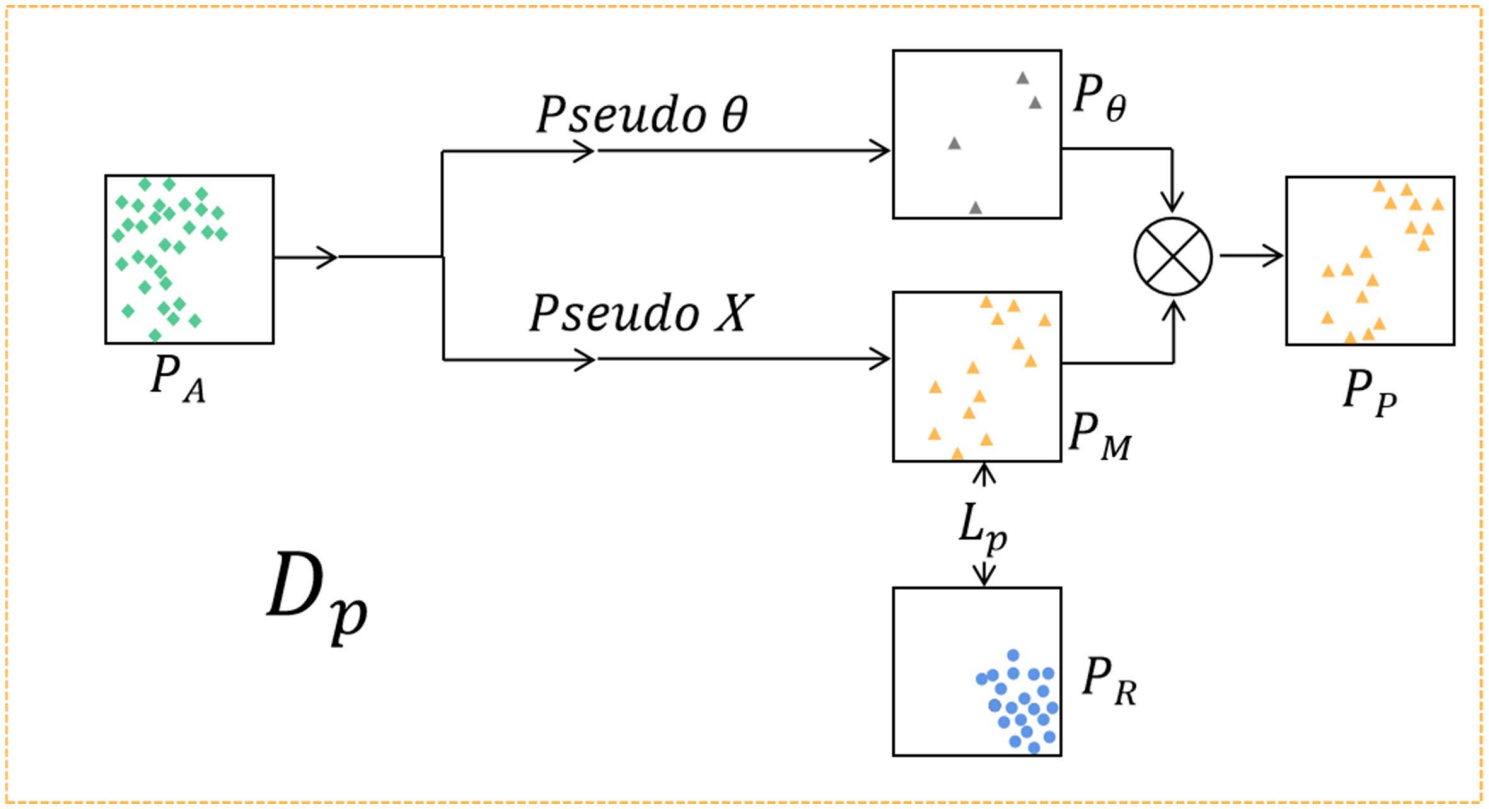
The pseudo-negative feature generator $D_{p}$. The $P_{A}$ of attack features and the $P_{R}$ of *bona fide* features are input into $D_{p}$. Firstly, according to $P_{A}$, the random generator is used to generate the $P_{M}$ that fits the distribution of $P_{A}$, and the loss function $L_{p}$ is designed to optimize the distribution $P_{M}$ of generated pseudo-negative features. To prevent overfitting of the data, a random noise $P_{\theta }$ is generated according to $P_{A}$, and the final virtual feature distribution $P_{P}$ is obtained by combining $P_{\theta }$ with $P_{M}$.

During the feature generation process, the corresponding feature distributions are computed by leveraging the extracted features from both attack and *bona fide* images. Then, the distribution data is fed into the data generator $D_{p}$, which uses a random data generator based on these distributions to generate a pseudo-negative feature distribution $P_{P}$ that fits the attack feature distribution. The structure of the data generator $D_{p}$ is presented in Figure 3.

This section introduces the proposed method from three aspects: feature analysis, feature generation, and loss function.

### 3.1 Feature analysis

**Figure 4:**
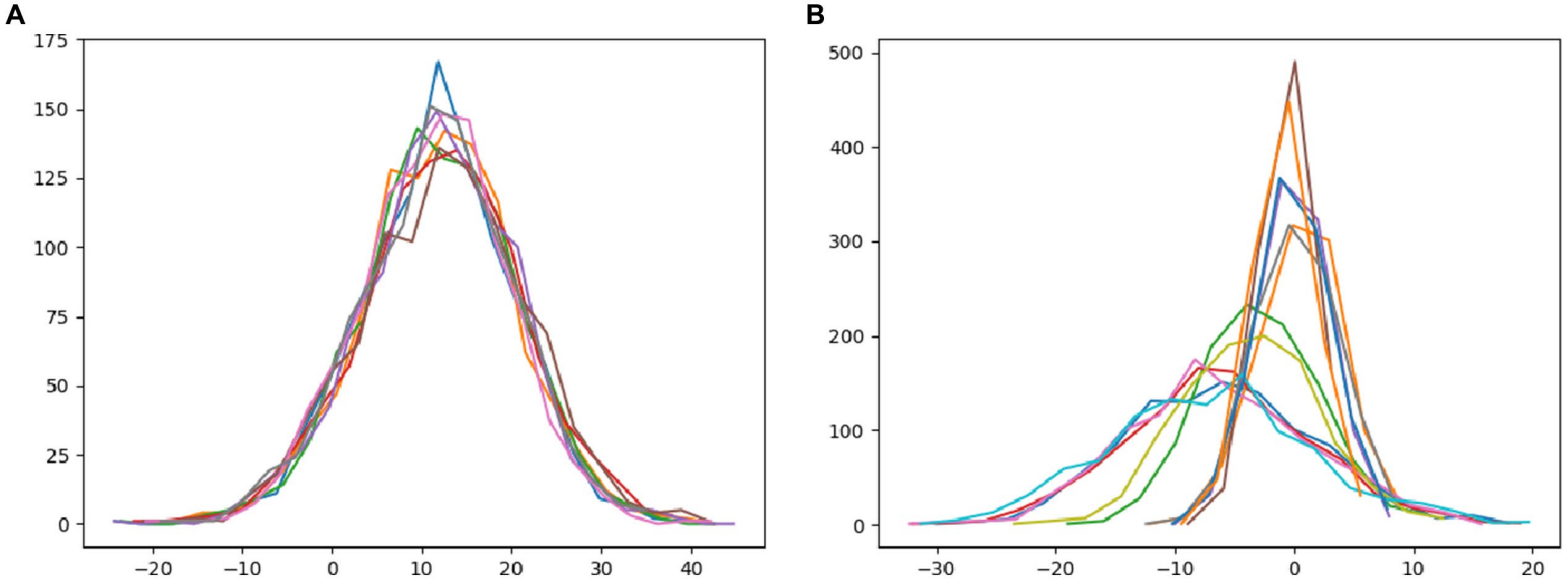
The distribution of the feature tensors of the statistical images. **(A)** The statistical tensor distribution of *bona fide* images of different types, and **(B)** the tensor of all types of attack images in the statistical dataset.

In this paper, we utilize Android and laptop camera devices to acquire face images and subsequently calculate their feature distributions, aiming to analyze the disparities between real and attack face images. As depicted in Figure 4A, it is evident that regardless of the capturing device used, the features of *bona fide* face images conform to a normal distribution, resulting in a relatively clustered pattern. Figure 4B illustrates the image features of attack faces across three distinct display media: three variations of iPad replay video attacks, iPhone replay video attacks, and photo print attacks. Notably, the feature distribution of attack face images employing different display media appears scattered, highlighting the variations in feature distribution among diverse attack methodologies.

In light of the characteristics of normal distribution, we aim to generate pseudo-negative feature data from the original sample feature data in order to enhance network performance. Toward this objective, our paper proposes a methodological framework. Initially, we examine the extracted feature data from the training samples obtained via Convolutional Neural Networks (CNNs). Subsequently, we synthesize pseudo-negative feature data that closely resembles the original sample feature data, ensuring alignment with the inherent distributional properties. Finally, we incorporate this pseudo-negative feature data into the classifier training process, with the ultimate goal of bolstering the accuracy and generalization capabilities of the face anti-spoofing system.

**Figure 5:**
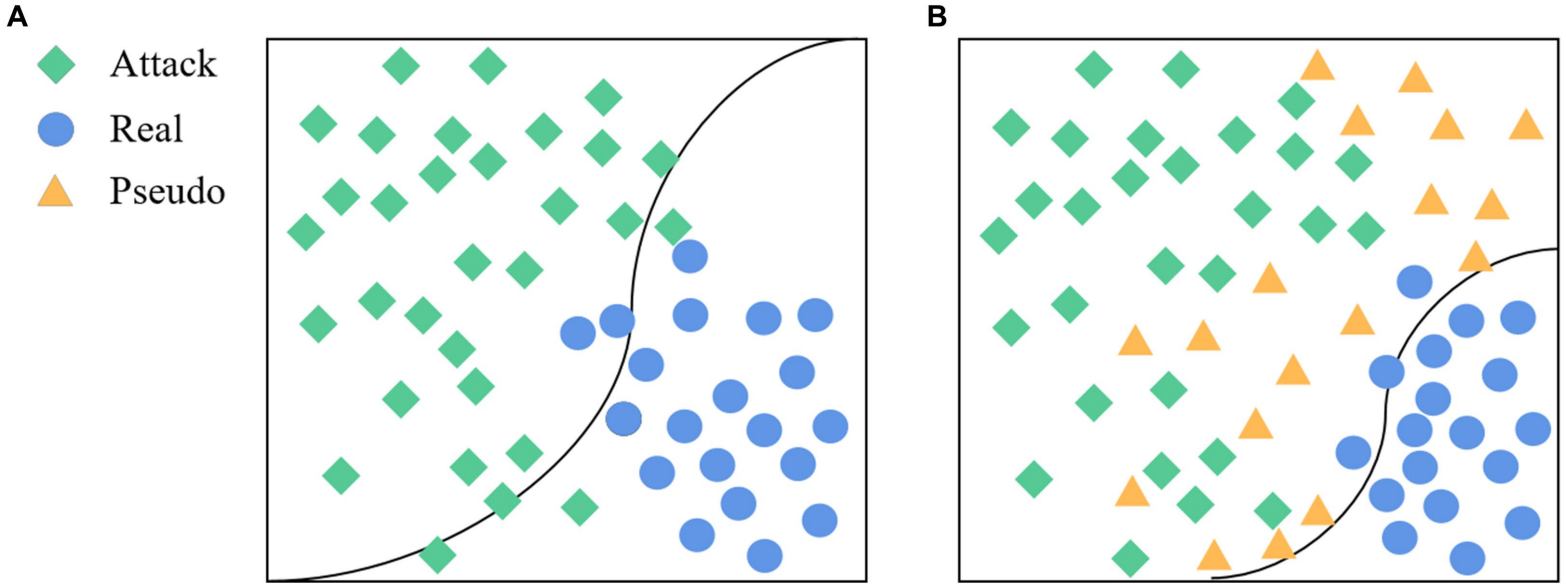
The goal of the proposed method. **(A)** The classification boundary without adding pseudo-negative features, and **(B)** the classification boundary after adding pseudo-negative features.

In face anti-spoofing systems, *bona fide* sample data are typically acquired through equipment-based face data collection. Conversely, attack samples, encompassing image-based and video replay assaults, primarily initiate with frontal face information gathering followed by secondary imaging involving facial prostheses via shooting equipment. Notably, while the *bona fide* sample collection method remains consistent across various data sets, attack samples may exhibit a more scattered distribution due to disparities in devices and attack methodologies (Jia et al., 2020). This difference makes the real face features of different data sets more likely to gather than the attack face features. In the practical application of the face anti-spoofing system, the classification boundary trained based on existing datasets may lead to overlapping characteristics between *bona fide* and novel attack sample data in certain domains, thereby impeding accurate classification. As illustrated in Figure 5A, the classification boundary delineates the feature space into *bona fide* and attack regions. To enhance system performance and ensure robust responsiveness to emerging attacks encountered in real-world scenarios, this study introduces the generation of pseudo-negative feature data (depicted in Figure 5B). This approach serves to augment the feature representation of samples, facilitating the clustering of *bona fide* data and optimizing classification outcomes. Consequently, the accuracy and generalization capabilities of face anti-spoofing systems are substantially improved.

### 3.2 Feature generation

In terms of current technology, the collection method for real face data across various datasets is relatively straightforward, as the equipment gathers facial data information directly. Consequently, the feature information of attack face samples tends to be more scattered compared to *bona fide* faces. Additionally, in practical applications, numerous unseen novel attack methods will arise. Therefore, the feature generation module performs feature generation and completes the new attack features in the unknown domain.

According to the analysis presented in section 3.1, the proposed image features follow a normal distribution, and the mean value and standard deviation can be calculated. In this study, a feature sequence that matches the mean and standard deviation of the original feature is randomly generated. Assuming $P_{R}$ is the distribution of the *bona fide* sample data, $P_{A}$ is the distribution of the attack sample data, and $P_{P}$ is the distribution of the generated features. To make the model achieve better performance, relative entropy, also known as Kullback–Leibler divergence, is used as the loss function of the feature-generating module. In the initialization process, $P_{P}=P_{A}$, i.e., the generated features and the attack sample features remain in the same distribution. At this time, the $D_{KL}\left(P_{P}∥P_{R}\right)$ has the minimum value, and the classification problem is relatively simple. In the optimization process, the distribution of pseudo-negative features approaches the *bona fide* sample gradually, which increases the multiformity of the attack sample, promotes the gathering of *bona fide* features, improves the classification accuracy of the face anti-spoofing system, and enhances the generalization of invisible new attacks. The loss function of feature generation is shown in the following Equation (1).


(1) $$L_{\text{Pseudo}}=\frac{D_{KL}\left(P_{P}∥P_{R}\right)}{D_{KL}\left(P_{P}∥P_{A}\right)+D_{KL}\left(P_{P}∥P_{R}\right)}$$

As shown in Equation (2), where $X_{A}$ represents the tensor data of the attack sample extracted by the feature extractor, $X_{i}$ randomly generates the data according to the mean and variance of the attack and the *bona fide* sample tensor, and $X_{\theta }$ represents the random noise generated according to the $D_{p}$.


(2) $$\begin{array}{c} D_{p}=\frac{1}{N}\sum_{i=1}^{N}\underset{i}{\min}∥X_{A}-X_{i}{∥}^{2}+X_{\theta } \end{array}\;$$

### 3.3 Loss function

**Figure 6:**
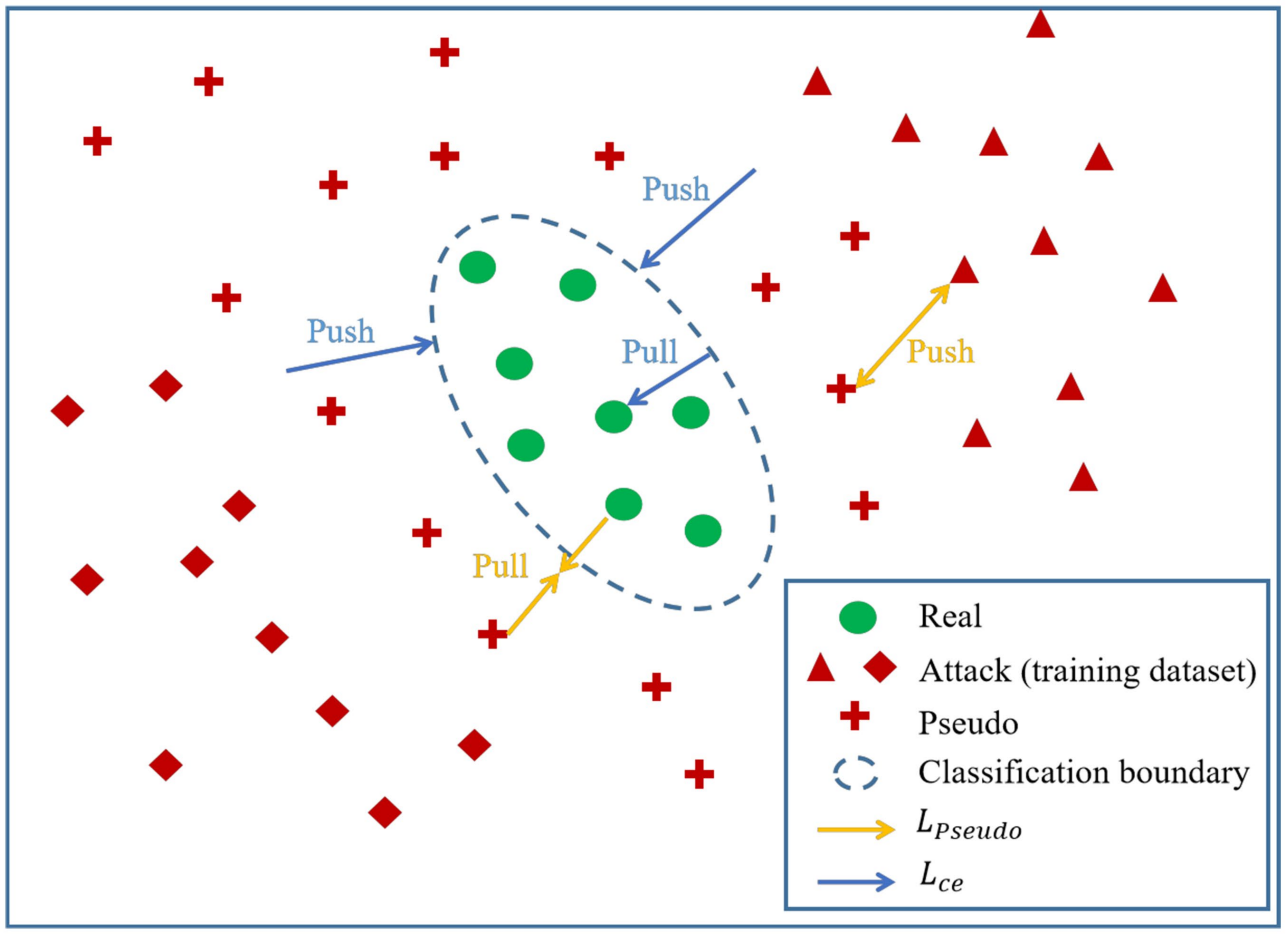
The visual representation of the roles played by $L_{ce}$ and $L_{\text{Pseudo}}$ in the processes of feature generation and classifier boundary training.

After generating the pseudo-negative feature data, it should be integrated into the face anti-spoofing system to enhance its performance. The cross-entropy loss function can be employed in neural networks as a metric to assess the similarity between the distribution of *bona fide* markers and the distribution predicted by the trained model. In this study, both the original feature data and the generated pseudo-negative feature data are concurrently fed into the loss function, aiming to enhance the generalizability and stability of the face anti-spoofing system in real-world applications. The overall network loss is defined as Equation (3):


(3) $$\begin{array}{c} L_{\text{Whole}}=\vartheta_{1}L_{ce}+\vartheta_{2}L_{\text{Pseudo}} \end{array}$$

where $L_{\text{Whole}}$ represents the overall loss function of the network, $L_{ce}$ represents the loss function of the original features, $\vartheta_{1}$ denotes the weight parameter of the original features, $L_{\text{Pseudo}}$ is the loss function of the newly generated features, and $\vartheta_{2}$ denotes the weight parameter of the newly generated features. The visual representation of the roles played by $L_{ce}$ and $L_{\text{Pseudo}}$ in the processes of feature generation and classifier boundary training is depicted in Figure 6.

## 4 Experimental setup

### 4.1 Databases

To evaluate the effectiveness of the proposed algorithm, it was tested on three publicly available face datasets, including MSU-MFSD (Wen et al., 2015), OULU-NPU (Boulkenafet et al., 2017a), and Replay-Attack (Chingovska et al., 2012).

**Figure 7:**
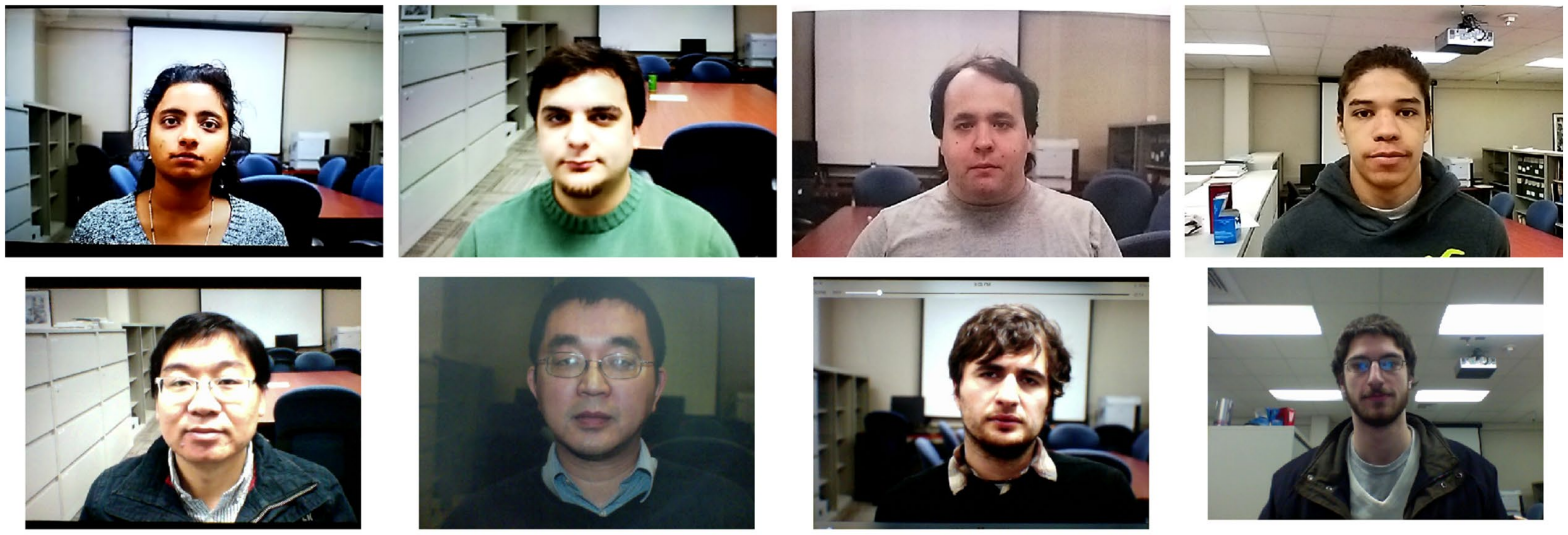
Some samples of the subjects recorded in the MSU-MFSD dataset. Images reproduced with permission from MSU-MFSD dataset (Wen et al., 2015).

The MSU-MFSD dataset (shown in Figure 7) was released by Michigan State University in 2015. Currently, it consists of 280 videos, publicly available and featuring 35 individuals. The dataset consists of three attack types: iPad air video replay attack, iphone5S video replay attack, and A3 paper printed photo attack.

**Figure 8:**
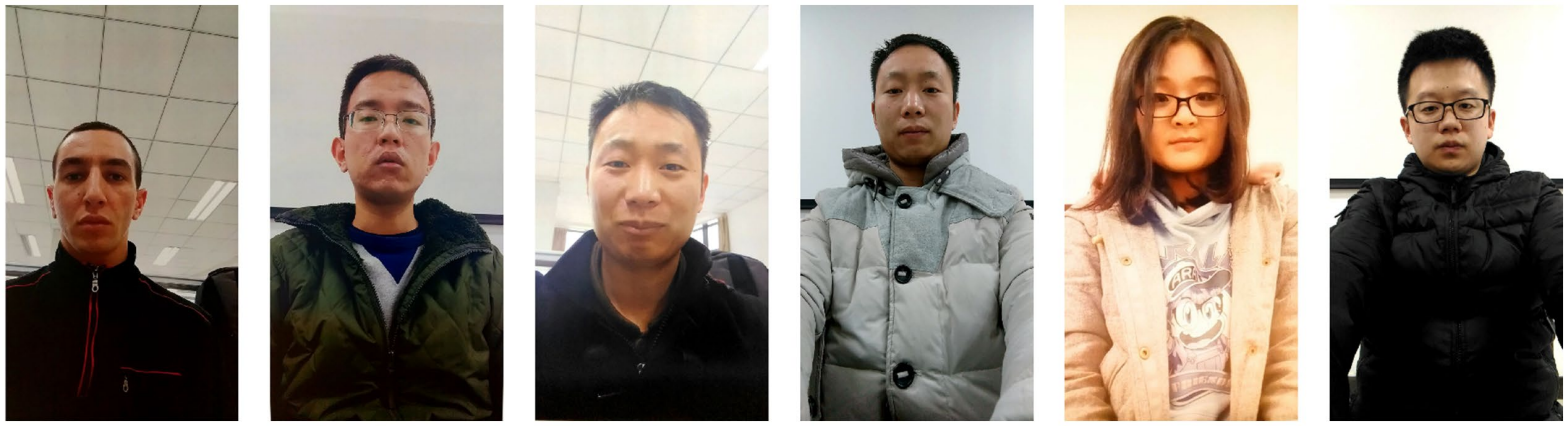
Some samples of the subjects recorded in the OULU-NPU dataset. Images reproduced with permission from OULU-NPU dataset (Boulkenafet et al., 2017a).

The OULU-NPU dataset (shown in Figure 8) was released by the University of Oulu in Finland in 2017. It consists of 4,950 video clips, captured from 55 participants with 90 videos collected per participant. The dataset consists of four types of attacks: photo attacks printed by two different printers, and video replay attacks displayed by two different display devices.

**Figure 9:**
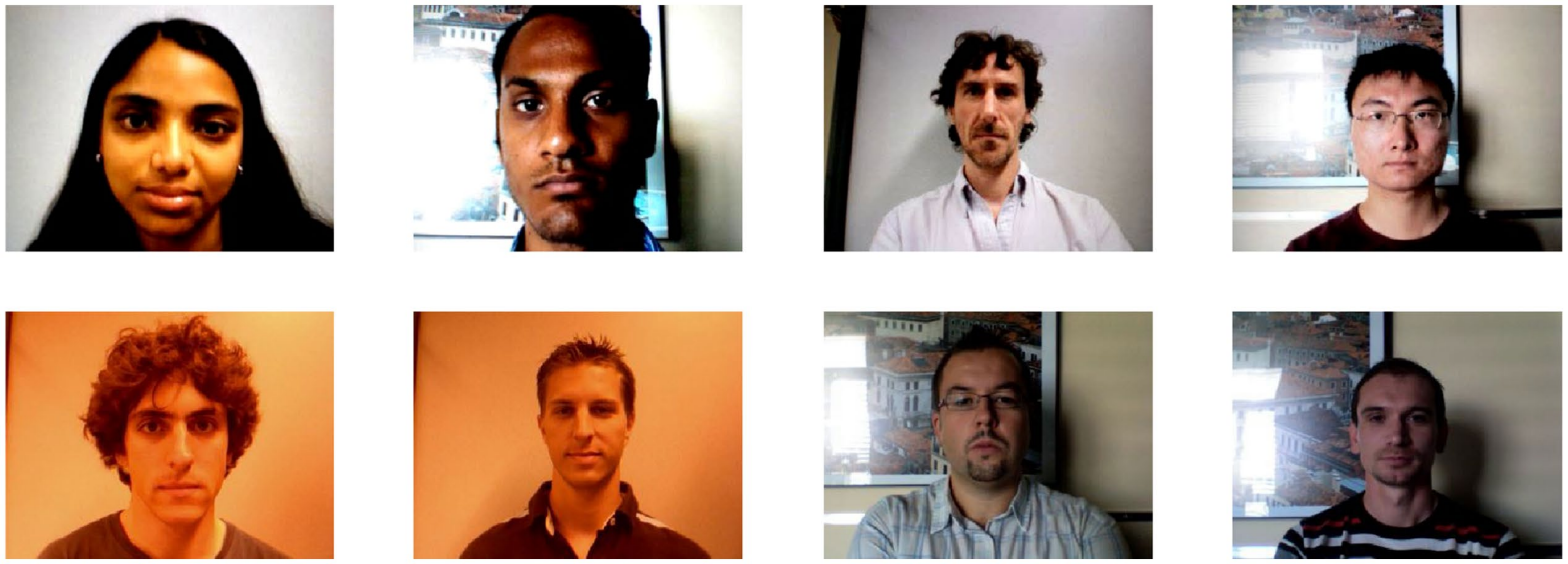
Some samples of the subjects recorded in the Replay-Attack dataset. Images reproduced with permission from Replay-Attack dataset (Chingovska et al., 2012).

The Replay-Attack dataset (shown in Figure 9) was released in 2017 and is comprised of 1,200 video clips. These videos feature 50 clients and showcase attack attempts under varying lighting conditions.

Since the dataset comprises entirely of video files, all videos and images were extracted frame-by-frame, and all images have undergone normalization. In these datasets, there are more attack samples than *bona fide* samples, with a large difference in number. During the training process, the quantity of attack and *bona fide* samples was carefully balanced to maintain a similar range, aiming to minimize both data quantity and the chance of overfitting. During data set division, owing to the varied nature of attack samples, the quantity of data samples gathered within identical environmental conditions was two to four times higher compared to *bona fide* samples. Therefore, the attack sample takes the image by the proportion of the *bona fide* sample. In contrast, the attack sample is often intercepted to maintain the amount of the two data in a similar range.

### 4.2 Experimental metrics

In face anti-spoofing, there are four types of prediction results: True Positives (*TP*), where positive samples are predicted by the model as positive classes; True Negatives (*TN*), where negative samples are predicted by the model as negative classes; False Positives (*FP*), where negative samples are predicted by the model as positive classes; False Negatives (*FN*), where positive samples are predicted by the model as negative classes.

Performance evaluation indicators include Attack Presentation Classification Error Rate (*APCER*), *Bona Fide* Presentation Classification Error Rate (*BPCER*), Average Classification Error Rate (*ACER*), Half Total Error Rate (*HTER*), and Area Under the ROC Curve (*AUC*). These performance indicators are calculated as follows Equations (4–7):


(4) $$\begin{array}{c} \text{APCER}=\frac{FP}{TN+FP} \end{array}\;$$

(5) $$\begin{array}{c} \text{BPCER}=\frac{FN}{TP+FN} \end{array}\;$$

(6) $$\text{ACER}=\frac{\text{APCER}+\text{BPCER}}{2.0}\;$$

(7) $$\text{HTER}=\frac{\text{FAR}+\text{FRR}}{2.0}\;$$

where *FAR* represents the false acceptance rate, and it is calculated as FAR=FP/(FP+TN), and *FRR* represents the false rejection rate, and it is calculated as FRR=FN/(FN+TP).

### 4.3 Experimental environment

The experiment was conducted on a computer equipped with an AMD Ryzen 75,800× 8-Core CPU, 32 GB memory, and Nvidia GTX 3060 GPU (12 GB video memory), and the computer runs the Windows 10 operating system. The proposed algorithm was implemented based on the PyTorch framework. The Adam optimizer was adopted for model optimization with a learning rate of 2.00e-4 and a batch size of 32.

## 5 Experimental results

### 5.1 Control experiment

In this paper, as a control group, the deep learning network AlexNet was trained and tested on the OULU-NPU dataset and MSU-MFSD dataset (Krizhevsky et al., 2012). Based on the native AlexNet, a pseudo-negative feature generation module was added, and then the model was trained and tested on two datasets. The performance of the two models on the OULU-NPU and MSU-FASD datasets is presented in Tables 1, 2, respectively. The results in the two tables show that in the model with the pseudo-negative feature generation module, APCER significantly decreased; in most protocols, BPCER reduced correspondingly, and the overall ACER was diminished.

**Table 1:** The performance on the OULU-NPU dataset.

Protocol	Model	APCER (%)	BPCER (%)	ACER (%)
I	AlexNet	0.94	79.90	40.42
	AlexNet+our	0.01	63.19	31.60
II	AlexNet	14.46	6.78	10.62
	AlexNet+our	5.06	10.46	7.76
III	AlexNet	3.40 ± 2.98	11.56 ± 7.58	7.17 ± 3.72
	AlexNet+our	2.33 ± 2.33	9.75 ± 5.25	6.04 ± 1.45
IV	AlexNet	9.07 ± 9.07	58.87 ± 33.87	32.84 ± 16.00
	AlexNet+our	3.53 ± 3.53	55.88 ± 25.88	29.71 ± 11.17

**Table 2:** The performance on the MSU-FASD dataset.

Model	APCER (%)	BPCER (%)	ACER (%)
AlexNet	1.47	5.27	3.37
AlexNet+our	1.39	3.99	2.69

### 5.2 Experimental discussion

The experiment evaluated the performance of the intra-test and inter-test. Specifically, the training and testing were performed on the same dataset, which can reflect the performance of the algorithm; cross-datasets indicate that the training set and test set are from different data sets, and the test on these datasets can usually reflect the generalization ability of the algorithm.

The experiments first compared the results of fusing different features on two datasets, followed by comparing the results of different fusion methods on two datasets, then compared the proposed method with some popular methods, and finally evaluated performance across databases on two datasets. The experimental results demonstrated the effectiveness of the proposed face detection method in face anti-spoofing.

The following four experiments were set for comparison in Table 3. Since there are four protocols in the OULU-NPU dataset, protocol 2 was selected based on the features of the MSU-MFSD dataset.

**Table 3:** Comparison of the experimental results.

Experiment	MSU-MFSD			OULU-NPU		
	APCER (%)	BPCER (%)	ACER (%)	APCER (%)	BPCER (%)	ACER (%)
1	1.47	5.27	3.37	14.46	6.78	10.62
2	1.39	3.99	2.69	5.06	10.46	7.76
3	20.71	65.23	42.97	25.36	45.82	35.59
4	20.07	65.97	43.02	7.29	35.41	21.35

Experiment 1: AlexNet networks without the pseudo-negative feature generator were tested with an intra-test on the OULU-NPU and MSU-MFSD datasets.

Experiment 2: AlexNet networks with the pseudo-negative feature generator were tested with an intra-test on the OULU-NPU and MSU-MFSD datasets.

Experiment 3: AlexNet networks without the pseudo-negative feature generator were tested with an inter-test on the OULU-NPU and MSU-MFSD datasets.

Experiment 4: AlexNet networks with the pseudo-negative feature generator were tested with an inter-test on the OULU-NPU and MSU-MFSD datasets.

To evaluate the effectiveness of our method, in Table 4, the OULU-NPU dataset was used to train and test the AlexNet and AlexNet+our (AlexNet network using the pseudo-feature generator), respectively, and the performance evaluation metrics were calculated. The results indicated that the proposed method achieved comparable performance with state-of-the-art methods (LBP + SVM, GRADIANT, and MILHP). We tested our model on the Replay-Attack dataset, as shown in Table 5. Compared with the state-of-the-art methods from the past 3 years (RGB+LBP and multilevel+ELBP), our model achieved superior performance in terms of accuracy and other evaluation metrics.

**Table 4:** Comparable performance on the OULU-NPU dataset.

Protocol	Model	APCER (%)	BPCER (%)	ACER (%)
I	LBP+SVM (George and Marcel, 2019)	12.9	51.7	32.3
	GRADIANT (Boulkenafet et al., 2017b)	1.3	12.5	6.9
	MILHP (Lin et al., 2018)	8.3	0.8	4.6
	AlexNet	0.9	79.9	40.4
	AlexNet+our	0.0	63.2	31.6
II	LBP+SVM (George and Marcel, 2019)	30.0	20.3	25.1
	GRADIANT (Boulkenafet et al., 2017b)	3.1	1.9	2.5
	MILHP (Lin et al., 2018)	5.6	5.3	5.4
	AlexNet	14.5	6.8	10.6
	AlexNet+our	5.06	10.46	7.76
III	LBP+SVM (George and Marcel, 2019)	28.5 ± 23.1	23.3 ± 18.0	25.9 ± 11.3
	GRADIANT (Boulkenafet et al., 2017b)	2.6 ± 3.9	5.0 ± 5.3	3.8 ± 2.4
	MILHP (Lin et al., 2018)	1.5 ± 1.2	6.4 ± 6.6	4.0 ± 2.9
	AlexNet	3.4 ± 3.0	11.6 ± 7.6	7.2 ± 3.7
	AlexNet+our	2.3 ± 2.3	9.8 ± 5.3	6.0 ± 1.5
IV	LBP+SVM (George and Marcel, 2019)	41.67 ± 27.03	55 ± 21.21	48.33 ± 6.07
	GRADIANT (Boulkenafet et al., 2017b)	5.0 ± 4.5	15.0 ± 7.1	10.0 ± 5
	MILHP (Lin et al., 2018)	15.8 ± 12.8	8.3 ± 15.7	12.0 ± 6.2
	AlexNet	9.1 ± 9.1	58.9 ± 33.9	32.8 ± 16.0
	AlexNet+our	3.5 ± 3.5	55.9 ± 25.9	29.7 ± 11.2

**Table 5:** Comparable performance on the Replay-Attack dataset.

Model	HTER(%)	EER(%)
RGB+LBP (Antil and Dhiman, 2023)	4.58	9.69
Multilevel+ELBP (Antil and Dhiman, 2022)	0.00	0.00
Dropblock (Wu et al., 2021)	0.29	0.00
Our	0.00	0.00

As shown in Table 3, the APCER of the AlexNet using a pseudo-negative feature generator decreased significantly on both within-set and cross-set tests, and BPCER also decreased, with only a few parts increasing slightly. The comparison results in Table 4 show that on the OULU-NPU dataset, the performance of AlexNet is not outstanding, and there is a significant performance gap with the mainstream methods. In contrast, the AlexNet using a pseudo-negative feature generator showed good performance in training and testing. The APCER and BPCER were significantly improved compared with those of AlexNet, and they were close to the performance evaluation indicators of mainstream methods.

To test the model’s generalization performance, cross-dataset testing was conducted on the MSU-MFSD dataset (referred to as M), OULU-NPU dataset (referred to as O), Replay-Attack dataset (referred to as R), and CASIA-FASD dataset (referred to as C; Zhang et al., 2012). Then, the results were compared with those of other mainstream experiments, as shown in Table 6. To further verify the performance of the model, we reduced the data set used for training. The experimental results are shown in Table 7. From Table 7, it can be observed that, when using a smaller dataset, our method can achieve results close to or even surpass those obtained from training on larger datasets.

**Table 6:** Comparison of the results between our experiment and the state-of-the-art in cross-domain face anti-spoofing detection.

Methods	O&C&R-to-M		O&M&R-to-C		O&C&M-to-R		R&C&M-to-O	
	ACER(%)	AUC(%)	ACER(%)	AUC(%)	ACER(%)	AUC(%)	ACER(%)	AUC(%)
MADDG (Shao et al., 2019)	17.69	88.06	24.50	84.51	22.19	84.99	27.89	80.02
ANRL (Liu et al., 2021b)	10.83	96.75	17.85	89.26	16.03	91.04	15.67	91.90
SSAN (Wang et al., 2022b)	6.67	98.75	10.00	96.67	8.88	96.79	13.72	93.63
Our	7.12	98.06	11.54	99.21	3.88	98.17	8.36	98.78

**Table 7:** Comparative cross-dataset testing results for similar models.

Experiment	Model	Train(videos)	HTER(%)	AUC(%)
M to R	Multilevel+ELBP (Antil and Dhiman, 2022)	280	24.3	-
M to R	Our	280	21.10	92.36
R&M to O	SSDG (Jia et al., 2020)	1,480	36.01	66.88
R&M to O	D^2^AN (Chen et al., 2021)	1,480	27.70	75.36
R&M to O	DRDG (Liu et al., 2021a)	1,480	33.35	69.14
R&M to O	ANRL (Liu et al., 2021b)	1,480	30.73	74.10
R&M to O	SSAN (Wang et al., 2022b)	1,480	29.44	76.62
M to O	Our	280	26.24	83.77

### 5.3 Feature distribution

**Figure 10:**
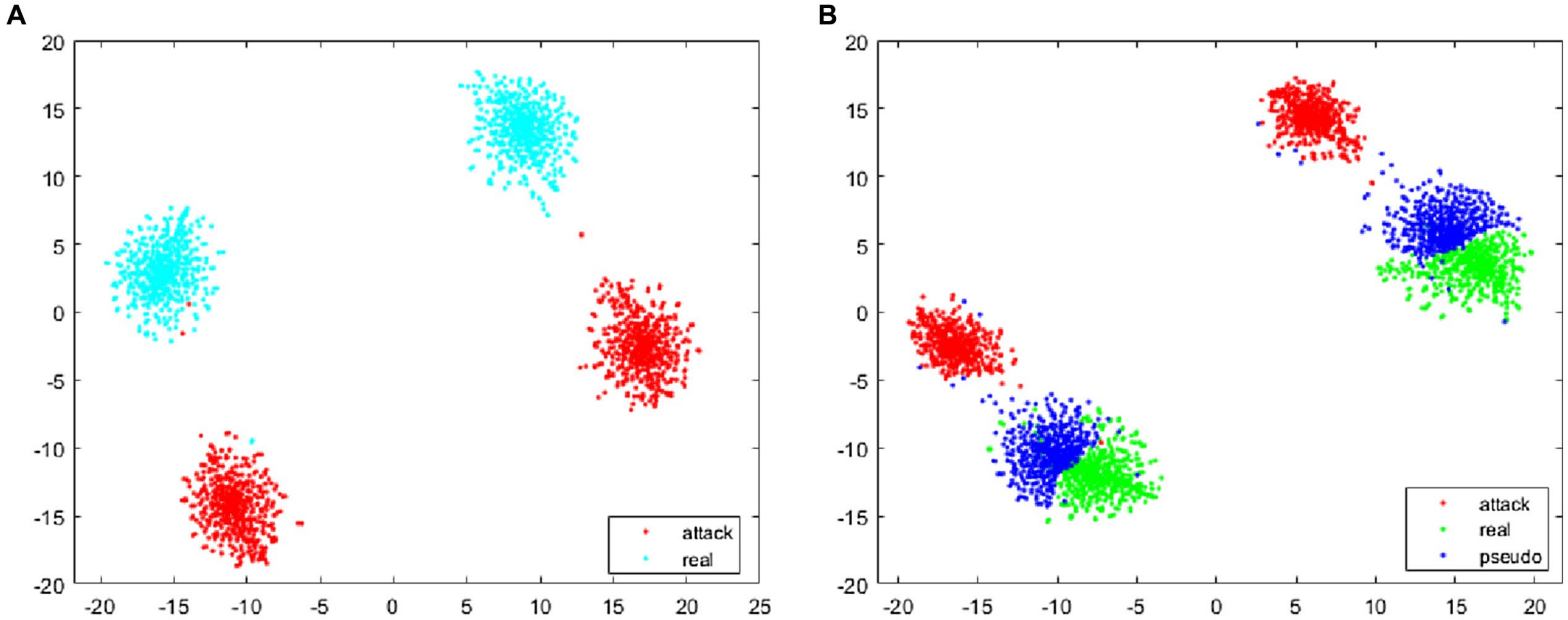
The feature cosine distance of images during training. **(A)** The training without using pseudo-negative features, **(B)** the training using pseudo-negative features.

The feature visualization algorithm was utilized to extract and compute the features of the training images, whose cosine distance is depicted in Figure 10. Specifically, Figure 10A presents the distance between the attack and the *bona fide* samples in the training phase. It can be seen that there is a large distance between the *bona fide* samples and the attack samples, and there are many blank unknown regions between the two types of samples. Since the face anti-spoofing system in practical applications may encounter some new attack data that did not appear in training, this paper generated false negative samples between the *bona fide* and attack samples. As shown in Figure 10B, the pseudo-negative samples are closer to the *bona fide* samples, indicating that the classification boundary of the face anti-spoofing system, during training, is more biased toward the *bona fide* samples. In practical applications, the face anti-spoofing system can achieve a good identification effect for new attacks that have not appeared in the dataset.

## 6 Conclusion

In this paper, a face anti-spoofing algorithm is proposed based on generated pseudo-negative features. Through continuous iteration, the original face anti-spoofing system achieves higher accuracy and robustness. Meanwhile, by adding pseudo-negative features, good results have been obtained in detecting attack samples. It shows that adding pseudo-negative class features enables the model to detect negative samples, and this affects the detection of positive examples in some cases. In this study, by constantly adjusting the strategy, new features are continually generated based on the image’s original features. Concurrently, a face anti-spoofing system is devised to counter emerging attacks within the feature space, resulting in the development of more effective strategies. Furthermore, this study promotes aggregation among *bona fide* examples while increasing scatter among attack examples, consequently bolstering the model’s robustness in unfamiliar territories. In future work, we will focus on eliminating the influence on positive examples to improve their detection effect.

## Data availability statement

Publicly available datasets were analyzed in this study. This data can be found at: Replay-Attack Database: https://www.idiap.ch/dataset/replayattack, MSU-MFSD Database: http://biometricscse.msu.edu/Publications/Databases/MSUMobileFaceSpoofing, and Oulu-NPU Database: https://sites.google.com/site/oulunpudatabase.

## Author contributions

YM: Writing – original draft, Writing – review & editing, Conceptualization, Data curation, Formal analysis, Funding acquisition, Methodology, Resources, Supervision, Validation. CL: Writing – original draft, Writing – review & editing, Data curation, Formal analysis, Investigation, Methodology, Software, Supervision, Validation. LL: Investigation, Resources, Supervision, Validation, Writing – original draft, Writing – review & editing. YW: Data curation, Investigation, Software, Supervision, Validation, Writing – review & editing. YX: Formal analysis, Investigation, Resources, Supervision, Validation, Writing – review & editing.
